# Supplementary material for: Machine-Learning-Based Detecting of Eyelid Closure and Smiling Using Surface Electromyography of Auricular Muscles in Patients with Postparalytic Facial Synkinesis: A Feasibility Study
Source: Diagnostics (Basel). 2023 Feb 2;13(3):554. doi: 10.3390/diagnostics13030554 (PMC9914547; doi:10.3390/diagnostics13030554)
Supplement: Supplementary file 1 [file diagnostics-13-00554-s001.zip › diagnostics-2005172-supplementary.pdf]

Supplement Table S1. Features for the machine learning EMG classification.

#	Feature	Equation	Reference
1	Mean Absolute Value	$\bar{x} = \frac{\sum_{i=0}^{N} x_i}{N}$	Hudgins et al. [27]
2	Variance	$var = \frac{\sum_{i=1}^{N} (x_i - \bar{x})^2}{N}$	Phinyomark et al. [28]
3	Zero Crossings	$\text{zc} = \sum_{i=1}^{N-1} [sgn(x_i \times x_{i+1})]$	[29]
4	Root Mean Square	$\text{RMS} = \sqrt{\frac{1}{N} \sum_{i=0}^{N} x_i^2}$	Phinyomark et al. [28]
5	Wave Form Length	$wl = \sum_{i=0}^{N-1} \lvert x_{i+1} - x_i \rvert$	Phinyomark et al. [28]
6	Cardinality	$c = card(\vec{x})$	Ortiz-Catalan [30]
7	Mean Teager–Kaiser Operator	$\overrightarrow{tk} = \begin{cases} x_i, & if\ i = 0\ or\ N \\ x_i^2 - (x_{i+1} \times x_{i-1}), & otherwise \end{cases}$ $\overline{tk} = \frac{\sum_{i=0}^{N} tk_i}{N}$	Li and Aruin [31]
8	Entropy	$H_{Sh} = -\sum_{i=1}^{16} p_i(\vec{x}) \log p_i(\vec{x})$	Shannon [32]
9	Modified Mean Frequency	$mmnf = \frac{\sum_{j=1}^{M} f_j A_j}{\sum_{j=1}^{M} A_j}$	Phinyomark et al. [28]
10	Mean Frequency	$mnf = \frac{\sum_{j=1}^{M} f_j P_j}{\sum_{j=1}^{M} P_j}$	Phinyomark et al. [28]
11	Feature Normalization	$F_{i,norm} = \frac{F_i - \overline{F}}{std(\vec{F})}$	Géron et al. [25]

List of the calculated features: $\vec{x}$... EMG vector of one window, x_i... i-th sample from $\vec{x}$, N... number of samples in $\vec{x}$, p_i ($\vec{x}$)... magnitude of the i-th bin when $\vec{x}$ is transformed into a 16-bin histogram based on the amplitude, f_j ... j-th frequency in the spectrum, M... number of discrete frequencies in the spectrum, A_j...amplitude of the frequency spectrum at f_j, P_j... amplitude of the power spectrum at f_j, $\vec{F}$… feature vector, F_i… i-th sample of $\vec{F}$, $F_{i,norm}$… normalized sample of $\vec{F}$, and $std(\vec{F})$… standard deviation of $\vec{F}$.
